# Cochlear implants in the United Kingdom: Awareness and utilization

**DOI:** 10.1179/1467010013Z.00000000077

**Published:** 2013-03

**Authors:** Chris Raine

**Affiliations:** Yorkshire Cochlear Implant Service, Bradford Royal Infirmary, Bradford, UK

**Keywords:** Cochlear implantation, History, Surgical access, Adults, Children, Incidence, Universal newborn hearing screening

## Abstract

**Introduction:**

Every child and adult in the United Kingdom who fulfils the criteria for cochlear implantation is entitled to receive treatment under the National Health Service (NHS); children since 2009 are eligible for bilateral simultaneous implants and adults single implants unless they have additional sensory needs.

**History:**

During a period between 1982 and 1990, when a number of individual teams ran programmes using charitable funding, the British Cochlear Implant Group approached the UK Department of Health, who agreed to set up a 4-year pilot study of 10 programmes, including one children's programme. The outcomes were collected and analysed by the Medical Research Council's Institute of Hearing Research. The results, showing positive outcomes for adults and children, were published in 1995 and subsequently funding was provided directly by the NHS.

**Access:**

Between 2001 and 2006 the Universal Newborn Hearing Screen (UNHS) was implemented in England and Wales and also in Scotland and Northern Ireland. Data from UNHS and also data from the three main cochlear implant manufacturers have allowed estimates of access to cochlear implants for children and adults within the criteria for implantation.

**Children:**

Between 2006 and 2011 the figures show that 74% of estimated eligible children aged 0–3 years have received implants and 94% by the age of 17.

**Adults:**

For adults the figures are considerably lower, with only about 5% of those eligible for an implant actually receiving one. The reasons for this include, to a lesser degree, the fact that guidelines by the National Institute of Clinical Excellence (NICE) are stricter than in some other European countries, but chiefly because of lack of awareness among candidates and professionals, both of criteria for eligibility and of the potential advantages from cochlear implantation.

## Introduction

In the United Kingdom (UK) all adults and children who fulfil the criteria for cochlear implantation (CI) are eligible for this treatment under the National Health Service (NHS). They are entitled to be assessed, implanted, and subsequently managed, entirely free of charge.

While CI is now an established and recognized treatment for patients with severe to profound sensorineural hearing loss it is relevant to appreciate the background as to how it was established in the UK. It is acknowledged that following Djourno and Eyries’ direct stimulation of the auditory nerve in Paris in 1957, significant strides were made in the USA, initially in Los Angeles and subsequently in San Francisco. Transformation from experimental treatment to actual clinical service took place when the US Food and Drug Administration (FDA) approved the use of the House-3M single channel implant system in 1984.

## History

In the UK the field of CI started from humble beginnings with basic research with external cochlear stimulation in 1978. Six patients were fitted with the External Pattern Input system funded by the Medical Research Council. This had a spring-loaded electrode positioned onto the promontory that stimulated the inner ear with an analogue signal from an in-the-ear removable device. The patients either had no tympanic membrane or had had the membrane surgically attached to the promontory.

## During the 1980s attitudes to CI in the UK were ‘conservative’

In 1982 the University College Hospital group (UCH) in collaboration with the University College of San Francisco (UCSF) implanted a multichannel device, sadly without much long-term success due to reliability factors. Subsequently, no further San Francisco devices were available, while the San Francisco team explored options for commercial manufacturing of their device, which eventually led to the *Advanced Bionics* implants. The UCH team during the 1980s started to use the Vienna single channel extra cochlear devices. Six patients were implanted before manufacturing stopped which stimulated the UCH team headed by Graham Fraser together with Michael Conway to approach the Department of Health and the Royal National Institute for Deaf People (RNID – now known as Action on Hearing Loss). From charitable funding Finetech manufactured 80 single channel UCH/RNID devices (Cooper *et al*., 1989). The team were able to demonstrate that their first 30 patients treated could gain considerable improvements in their lip reading and communication ability (Cooper *et al*., 1989). All acquired useful awareness of environmental sounds with improvement in their quality of life. None achieved open set without lip reading.

By 1985 the Nucleus 22-channel implant, designed by Graeme Clark in Melbourne, was commercially available and the Food and Drug Administration (FDA) of the USA approved its use for Adults in the same year. The use of these implants in the UK started in Manchester and Kilmarnock in 1988 with several centres commencing shortly after, with support from charitable sources.

Around this time the British Cochlear Implant Group (BCIG) was formed by members of the existing cochlear implant teams, in all disciplines. One of its initial functions was to attempt to persuade the UK Department of Health to fund cochlear implants in the UK. In spring 1990 the Department of Health invited bids from existing and potential service providers to bid for a 3-year centrally funded programme for CIs for adults and children, using the Nucleus 22 multichannel implant and with each programme implanting about 10 patients a year. The Medical Research Council's Institute of Hearing Research was commissioned to evaluate the services. The evaluation had three goals: (1) to establish if CI could be an effective routine treatment. (2) Assess initial benefits to young children and (3) derive recommendations for the future form and scale of services nationally. This ‘National Cochlear Implant Programme’ was also rolled out to Scotland in 1991 by the Home and Health Department in selected hospitals and by Health and Social Services in Northern Ireland in 1992.

In addition to the 10 funded programmes several teams continued to provide CI services by independent charitable funding.

Prior to the programme about 80 people had been implanted, of whom the majority had received the UCH/RNID device, most of the others the Nucleus 22-channel device.

At the end of the programme nearly 300 patients over 10 years of age had been implanted. It is interesting to note that despite the fact that some 60% of those with a profound hearing loss are aged 70 years or older yet only 6% of those implanted in the study were over 70 years (Davis, 1989). Various reasons may be ascribed to this – unwillingness to commit to evaluation, surgery and extensive rehabilitation. During selection it was recognized that physical fitness and intellectual ability were factors.

In 1990 the FDA approved the use of the Nucleus 22 in children 2–17 years. By 1994 111 children had been implanted within the UK.

The report from the Institute of Hearing Research: ‘Cochlear Implantation in the UK 1990–1994’ by Summerfield and Marshall (1995) was pivotal in showing that CI services delivered through multidisciplinary teams produced effective outcomes for both adults and children. There was also a ‘learning curve’ in patient assessment and selection, surgical complications and rehabilitation.

Following on from the 1990/1994 programmes, various funding streams were established through the NHS. The sources of funding were varied as the NHS went through structural changes. Some local purchasers had policies of not funding adults, others had strict allocations. This not only let to frustration but developed a potential backlog of patients potentially suitable for treatment.

**Figure 1:**
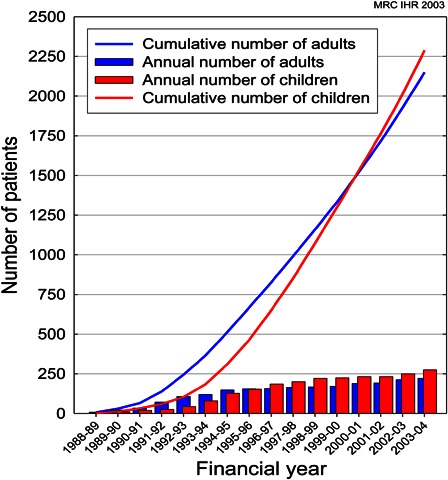
MRC IHR cumulative and annual data of cochlear implantation in children and adults (courtesy Prof. A Q Summerfield).

Annual numbers were initially collected by the Medical Research Council Institute of Hearing Research (MRC IHR). Fig. 1 shows the annual and cumulative figures for adults and children supplied to the MRC IHR by clinical coordinators of CI centres from 1988/1989 to 2003/2004.

Data were also collected by members of the National Cochlear Implant Users Association (NCIUA) Table 1 shows continued growth in the number of patients treated (NCUIA, 2010).

**Table 1:** CI surgical data collected by NCIUA

Year	Adults	Children
2005	239	332
2006	292	375
2007	350	429
2008	504	448
2009	498	527

CI is currently delivered through 17 NHS units in the UK.

The National Institute of Health and Clinical Excellence (NICE) initiated a technology appraisal process into the clinical and cost effectiveness of CI in adults and children. The review evaluated evidence on pre-lingually deafened children; post-lingually deafened children; and adults with acquired and progressive deafness and adults born deaf. The final appraisal document was published in February 2009 (TAG166, 2009).

Some of the main outcomes were:

- Unilateral CI is recommended as an option for people with severe to profound deafness who do not receive adequate benefit from acoustic hearing aids.
- Simultaneous bilateral CI is recommended as an option for the following groups of people with severe to profound deafness who do not receive adequate benefit from acoustic hearing aids:
  - children
  - adults who are blind or who have other disabilities that increase their reliance on auditory stimuli as a primary sensory mechanism for spatial awareness.

Sequential CI was not recommended as an option for people with severe to profound deafness. However, people who had a unilateral implant before publication of the guidance, and who fall into one of the categories described for bilateral implantation, should have the option of an additional implant in the other ear if this is considered to provide sufficient benefit by the responsible clinician after an informed discussion with the individual person and their carers.

The guidance also set clinical criteria for implantation for severe to profound deafness is defined as the ability to hear only sounds that are louder than 90 dB HL at frequencies of 2 and 4 kHz without acoustic hearing aids. Also the absence of adequate benefit from acoustic hearing aids, defined as follows:

- for adults, a score of 50% or greater on Bamford–Kowal–Bench (BKB) sentence testing at a sound intensity of 70 dB SPL;
- for children, speech, language, and listening skills appropriate to age, developmental stage, and cognitive ability.

The clinical criteria for CI in the UK are quite strict compared with some other countries. However, once a patient has been assessed by a multidisciplinary team funding has been relatively secure.

## Do patients with severe to profound hearing loss have access to CIs?

The most recent BCIG review of results was in November 2011 (Table 2).

**Table 2:** BCIG Survey November 2011

	Adults	Children	Total
How many unilateral CIs were you supporting on 31 March 2011?	4959	2525	7484
How many bilateral CIs were you supporting on 31 March 2011?	186	1259	1445
How many ‘new’ unilateral cochlear implant procedures were carried out in the past 12 months?	494	121	615
How many ‘new’ simultaneous CI procedures were carried out in the last 12 months?	14	276	290
How many sequential CI procedures were performed in the contralateral ear in the last 12 months?	14	258	272

Globally the figures show that a significant number of children now have access to bilateral implantation. Over the last couple of years significant additional workload has been placed on centres to review children with unilateral devices to see if they would be suitable for sequential surgery, especially as there appears to be evidence of a ‘critical age’ after which a second, sequential implant may be relatively ineffective for a congenitally deaf child who is already making good use of an implant in the original ear (Graham *et al*., 2009). This process in most centres has been completed.

While there are now several thousand patients with CIs we need to explore whether we are meeting clinical need.

## Children

To try and assess this, two sources of data were accessed. The first is the annual reported incidence of hearing loss as detected from the Universal Newborn Hearing Screen (UNHS). From 2001 to 2006 the Department of Health rolled out a programme of UNHS in England. Wales was fully implemented by 2004 and Scotland developed their service in approximately 2005. It has been recognized that such screening is efficient (Davis *et al*., 2001) and cost effective (Stevens *et al*., 1998).

Fortnum *et al*. (2001) assessed the levels of permanent childhood hearing impairment (PCHI) based on hearing levels >40 dB averaged over 0.5, 1, 2 and 4 kHz in children born from 1980 to 1995, resident in the UK in 1998. Prevalence rose from 0.91 (95% confidence interval 0.85 to 0.98) for 3 year olds to 1.65 (1.62 to 1.68) for children aged 9–16 years. A capture–recapture technique was used to estimate the extent of under-ascertainment in the population survey. Adjustment for under-ascertainment increased estimates to 1.07 (1.03 to 1.12) and 2.05 (2.02 to 2.08).

The prevalence of confirmed severe and profound PCHI was shown to uniformly increase until the age of 9 years (Fortnum *et al*., 2001).

The number of detected children born with a permanent hearing impairment of ≥40 dB in the better ear was supplied by the English and Welsh agencies. Using these data the prevalence for children with severe (71–95 dB HL) and profound (>95 dB HL) losses can be estimated. However, current NICE guidelines only allow implantation if hearing is ≥90 dB at 2 and 4 kHz. To try and ‘*estimate*’ a potential population suitable for CI surgery for 0–3 years and 0–16 years all the ‘profounds’ and 20% of the ‘severes’ were calculated from UNHS for England and Wales from 2006 to 2011.

To assess the rate of implantation by years, the three major implant companies Advance Bionics, Cochlear, and Med El were approached and they kindly supplied data on ‘Surgical Registrations’ (the numbers of people receiving implants) for the financial years 2006–2011 (Table 3). Patient registrations were allocated into the age groups at the time of surgery. Simultaneous implants were counted as one implant and sequential, second implants omitted; only the year in which the first implant was inserted was used. Data were supplied for the whole of the UK (Fig. 4).

**Table 3:** Registration data by age group for CI performed in England and Wales

	<3	3–17	18–29	30–49	50–64	65–80	>80	Unknown
2006	141	173	28	69	71	74	10	19
2007	147	179	40	99	89	69	5	30
2008	186	202	49	124	104	120	20	21
2009	182	180	35	−13	78	97	16	25
2010	161	178	54	134	105	103	25	41
2011	149	189	27	123	104	101	26	57

As UNHS data were not readily available for Scotland, surgical data from the only implant centre, in Kilmarnock, were kindly supplied and deducted from the total data. This allowed a comparison to be made in the data from England and Wales between the number of children with hearing loss likely to benefit from an implant and the number of children who were implanted.

**Figure 2:**
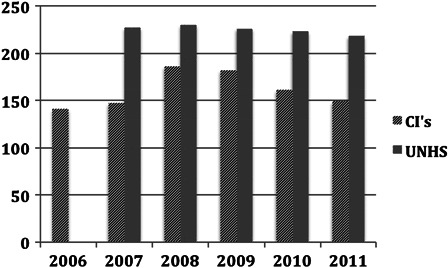
Estimate of the number of children (0–3 years) in England and Wales with severe to profound hearing loss and registered as having had CI performed.

The surgical registrations for the first 3 years of life in England and Wales were plotted against UNHS figures of children of a similar age (Fig. 2).

On average the rate of implantation was 74% of estimated eligible children with severe to profound hearing loss for this age group.

**Figure 3:**
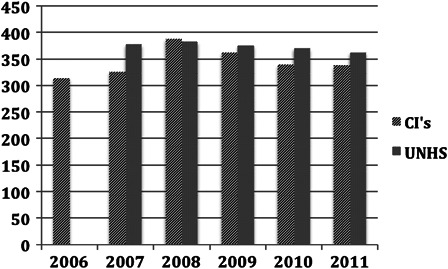
Estimate of the number of children (0–16 years) in England and Wales with severe to profound hearing loss and registered as having had CI performed.

Similarly, surgical registrations up to 17 years were plotted against UNHS figures for the cohort of children with estimated severe to profound loss for this age range (Fig. 3).

For the cohort 0–17 years the implantation rate on average was 94% of the estimated number of children with severe to profound hearing loss from UNHS.

## Adults

**Figure 4:**
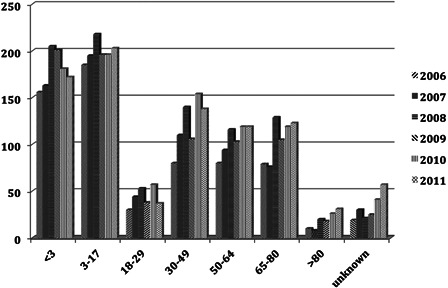
Surgical registration data by years for CI performed in the UK.

Adults only have access to a single CI unless they have additional sensory impairment.(TAG166, 2009). Prior to the NICE report, patients who became deaf following meningitis or ossifying conditions affecting the cochlea could usually have bilateral CI. Fig. 4 highlights the surgery performed within the UK between 2006 and 2011.

## Discussion

It is important to try and evaluate access to CIs as they have an important role in the management of patients with severe to profound hearing loss.

*For children:* the early recognition of PCHI by UNHS has been very important in the early detection and initiation of appropriate management. Fortnum *et al*. (2001) showed a prevalence of 1.07/1000 to the age of 3 with a better ear hearing loss ≥40 dB HL increasing to 2.05/1000 by 9–16 years. The prevalence of confirmed hearing impairment increases to 9 years, then levels out. Possible reasons for this include: non-diagnosis at screening; post-natal acquisition of hearing loss; late onset of progressive hearing impairment; and children now resident in the UK but born abroad.

The use of cochlear implants has steadily grown over the years. Clinical criteria have also changed over the years with recognition that early detection and implantation give superior results. In the UK, comprehensive clinical data on UNHS have been collected on children with PCHI ≥40 dB HL since the mid-2000s. Using data supplied by services in England and Wales the number of potential candidates with a hearing loss of ≥90 dB HL was estimated. During the years 2006–2011 the number of children identified was approximately 225 by 3 years, and 375 by 9–16 years. Assuming that the prevalence is constant, then those children <3 years of age who might have their implant at any time within a 3-year period were compared with the actual annual registrations of CI surgery. Using surgical data based on registration was thought to be the most accurate way of comparing use with anticipated use. The current rate of implantation in the 0–3 years of age was on average 74%. This increased during childhood to 93% by the time the children reached 17 years.

There appears (Fig. 2) to be an increasing rate of CI surgery in the under threes possibly due to the ‘roll out’ of UNHS. This improvement also predates the clinical approval by NICE in 2009 in treating children and adults with severe to profound deafness (TAG166, 2009). The impact of NICE on children has been the access to bilateral simultaneous CI for new patients and sequential implants for those implanted prior to 2009 after appropriate clinical assessment.

It may be logical that the rate of surgery is below and is parallel to the incidence of hearing impairment. Not all children are necessarily referred to a regional unit or are found to be suitable once assessment has been performed. Not all parents agree to referral. In the evaluation of the group 0–16 years the annual surgical registration is about 340 new cases and is approaching the anticipated average incidence of 370 representing about 93% of suitable children.

It is known that the incidence of acquired and progressive losses steadily rise to about 9 years of age then levels out. From MRC IHR data about 280 surgeries took place in 2003/2004. The NCIUA reported a significant rise from 2005 to 2009 (NCUIA, 2010) and what we might be seeing in reducing numbers are related to the catching up of a previous ‘backlog’. Again the ‘backlog’ may be due to various factors: detection, awareness, and improvement in funding. Clearly the current trends need to be monitored.

Continued clinical surveillance of children's hearing needs to be encouraged to allow earlier referral of children with progressive hearing losses to cochlear implant programmes.

*For adults*: the incidence of hearing impairment increases with age: 4.6% of 18–40 year olds are affected by a loss of at least 25 dB, this rises to 60% in 71–80 year olds (Shield, 2006). There is considerable variation in both qualitative and quantitative descriptions of deafness. Care must therefore be taken when considering or reviewing literature on any aspects of deafness where grades of hearing loss are discussed. NICE (TAG166, 2009) reported that there are approximately 613 000 people older than 16 years with severe to profound deafness in England and Wales. Davis's national study in 1995 reported that in 18–80 year olds 0.7% had a severe hearing loss (70–94 dB HL) and 0.2% a profound (>95 dB HL) (Davis, 1995). With a population of 51.4 million over 15 years of age in the UK (Office of National Statistics, 2011) there is an estimated 0.103 million with a profound loss and 0.36 million with a severe loss. It would be difficult to estimate how many adults in the severe group would be suitable for CI.

Cochlear implant units in the UK are currently supporting over 5000 adults. With annual data showing a slow but steady growth in numbers of adults implanted per year from about 240 in 2003/2004 to about 500 in 2010/2011. This would appear to be significantly below any predictions of need.

What factors might be affecting such a potential reduction in perceived access for adults in the UK?

The UK appears to perform half the number per million of population as compared with Germany or Austria (van Hardeveld, 2010). However, the clinical criteria for CI set by NICE set specific targets. With appropriate hearing aid provision a score of less than 50% on BKB sentence testing at 65 dB SPL in quiet, and Pure Tone thresholds of 90 dB or higher at 2 and 4 kHz are the criteria for eligibility. Clinically it is appreciated that this does not reflect the ‘real’ world as nobody speaks at such a high intensity. Testing in noise and assessment of performance with monosyllabic words would be more appropriate. Guidelines published on behalf of the Board of the German Society for Oto-Rhino-Laryngology, Head and Neck Surgery (http://www.acir.de/ADANO_ci_leitlinie.pdf, 2012) do not specify audiological criteria, giving the clinician possibly more clinical freedom. This would be a suitable task for BCIG to reassess and review in order to advise NICE when its criteria are next reviewed.

In many situations hearing loss is progressive in adults. Patients may have grown to accept their problems; families can support and reduce the effect of hearing loss. Patients accept deterioration as a natural ageing process and may be reluctant to undergo surgery.

General practitioners may not be aware of treatments but this should not be a barrier for referral to ENT/Audiology units. Sudden loss has significantly more impact, and is more likely to initiate a referral.

BCIG is currently embarking on a poster ‘awareness campaign’ targeting audiological units. It would also be appropriate for them to press for more representative tests of functional listening.

## Conclusion

This review raises more questions than it answers. What is required is closer evaluation of children detected by UNHS with moderate to profound hearing impairment to register with specialized audiology/CI units. Clearly we do not have sufficient data to address and understand the needs of the adult population. We also continue to collect accurate annual age-based data on surgery.

In the UK cochlear implants are available and free of charge for all UK residents, children and adults, who fulfil the NICE criteria for cochlear implants. The recent figures comparing rates of surgery against numbers of potentially suitable cases suggest that approximately 74% of suitable children aged 0–3 years of age have received cochlear implants, increasing to 94% of these children by the time they have reached 17 years of age. For adults the comparable figures suggest that only about 5% of the anticipated population of suitable adults receive cochlear implants, depending on what figures are used to estimate need. The next tasks will be to improve the figures for adults and to increase the proportion of eligible children receiving their implant before the age of 4.
